# Use of a novel cytotoxic HEXIM1 peptide in the directed breast cancer therapy

**DOI:** 10.18632/oncotarget.6794

**Published:** 2015-12-29

**Authors:** Shu Hui Neo, Qiao Jing Lew, Ser Mei Koh, Lu Zheng, Xuezhi Bi, Sheng-Hao Chao

**Affiliations:** ^1^ Expression Engineering and Bioprocessing Technology Institute, Agency for Science, Technology and Research (A*STAR), Singapore 138668; ^2^ Temasek Polytechnic, Singapore 529757; ^3^ Proteomics Groups, Bioprocessing Technology Institute, Agency for Science, Technology and Research (A*STAR), Singapore 138668; ^4^ Department of Microbiology, National University of Singapore, Singapore 117597

**Keywords:** HEXIM1, NPM, KLA, P-TEFb, p53

## Abstract

Hexamethylene bisacetamide-inducible protein 1 (HEXIM1) is best known as the inhibitor of positive transcription elongation factor b (P-TEFb) and is recently identified as a novel positive regulator of p53. We previously showed the basic region (BR) of HEXIM1 mediates the binding of HEXIM1 to a nucleolar protein, nucleophosmin (NPM), and can be ubiquitinated by human double minute 2 protein. Here we identify a cytotoxic peptide derived from the BR of HEXIM1. When fused with a cell-penetrating peptide, the HEXIM1 BR peptide triggers rapid cytotoxic effect independent of p53. Similarly, when the BR peptide is linked with a breast cancer cell targeting peptide, LTV, the LTV-BR fusion peptide exhibits specific killing of breast cancer cells, which is not observed with the commonly used cytotoxic peptide, KLA. Importantly, the BR peptide fails to enter cells by itself and does not induce any cytotoxic effects when it is not guided by any cell-penetrating or cancer targeting peptides. We showed that HEXIM1 BR peptide depolarizes mitochondrial membrane potential in a p53-dependent manner and its cell-killing activity is not suppressed by caspase inhibition. Furthermore, we observed an accumulation of the internalized BR peptide in the nucleoli of treated cells and an altered localization of NPM. These results illustrate a novel mechanism which the BR peptide induces cell death and can potentially be used as a novel therapeutic strategy against breast cancer.

## INTRODUCTION

HEXIM1 was originally identified from vascular smooth muscle cells treated with hexamethylene bisacetamide (HMBA), an anti-proliferation compound [1]. To date, HEXIM1 is best known as the inhibitor of positive transcription elongation factor b (P-TEFb), which controls the elongation phase of RNA polymerase II transcription and is required for transcriptional regulation of human immunodeficiency virus [2, 3]. HEXIM1 blocks the activity of P-TEFb only when associated with the 7SK small nuclear RNA (snRNA) through its basic region (BR), while neither 7SK snRNA nor HEXIM1 alone instigates any effects [4–7].

The potential involvement of HEXIM1 in cancers was first reported in 2003. HEXIM1 was found to associate with estrogen receptor α (ERα), which is widely targeted in breast cancer therapy. HEXIM1 exhibits its anti-cancer effects through the inhibition of the ERα-dependent gene expression [8]. Our previous studies have demonstrated the functional interactions between HEXIM1 and other critical proteins involved in cancer, including the tumor suppressor p53, human double minute-2 protein (HDM2), and nucleophosmin (NPM). HEXIM1 directly binds to p53 and stabilizes p53 by blocking the HDM2-mediated ubiquitination of p53. Furthermore, HEXIM1 is required for p53 activation induced by anti-cancer drugs/compounds [9, 10]. We have earlier identified six lysine residues located within the BR of HEXIM1 (*i.e*. Lys-150-152 and Lys 159-161) as the major sites for HDM2 ubiquitination. The HDM2-ubiquitinated HEXIM1 exhibits a stronger inhibition in P-TEFb-dependent transcription [11]. HEXIM1 also binds to NPM [12], a nucleolar protein which serves an important factor in ribosome biogenesis [13, 14] and has been implicated in DNA replication, transcription and repair [15]. About 35% of acute myeloid leukemia (AML) patients carry the cytoplasmic-misallocated mutant form of NPM, NPMc+ [16]. NPMc+ is found to interact and sequester a portion of HEXIM1 in the cytoplasm of the NPMc+ AML cell line and activates P-TEFb-dependent transcription, suggesting the involvement of HEXIM1 in tumorigenesis of AML [17].

A commercially available p53 activating peptide, which contains a cell-penetrating peptide fused with a p53 peptide, which contains the HDM2-ubiquitinated sites (*i.e*. a.a. 361–382), is reported to induce apoptosis in mutant and wild-type p53-bearing human cell lines [18]. The internalized p53 peptide is likely to compete with the endogenous p53 protein in binding to HDM2 and protect the endogenous p53 from HDM2-mediated ubiquitination, resulting in stabilization and activation of p53. We reason that the HEXIM1 BR may exhibit similar function by disrupting the p53-HDM2 interaction. In this study, we evaluate the potential use of the HEXIM1 BR peptide as the therapeutic peptide against breast cancer. Interestingly, we find that the HEXIM1 BR peptide induces potent and rapid cytotoxicity through a novel p53-independent mechanism. Our results show that HEXIM1 BR peptide exhibits specific cell killing when directed by a breast cancer targeting peptide, suggesting the use of the BR peptide in anti-tumor strategies.

## RESULTS

### HEXIM1 BR peptide induces cell death independently of p53, P-TEFb, and apoptosis *in vitro*

**Figure 1:**
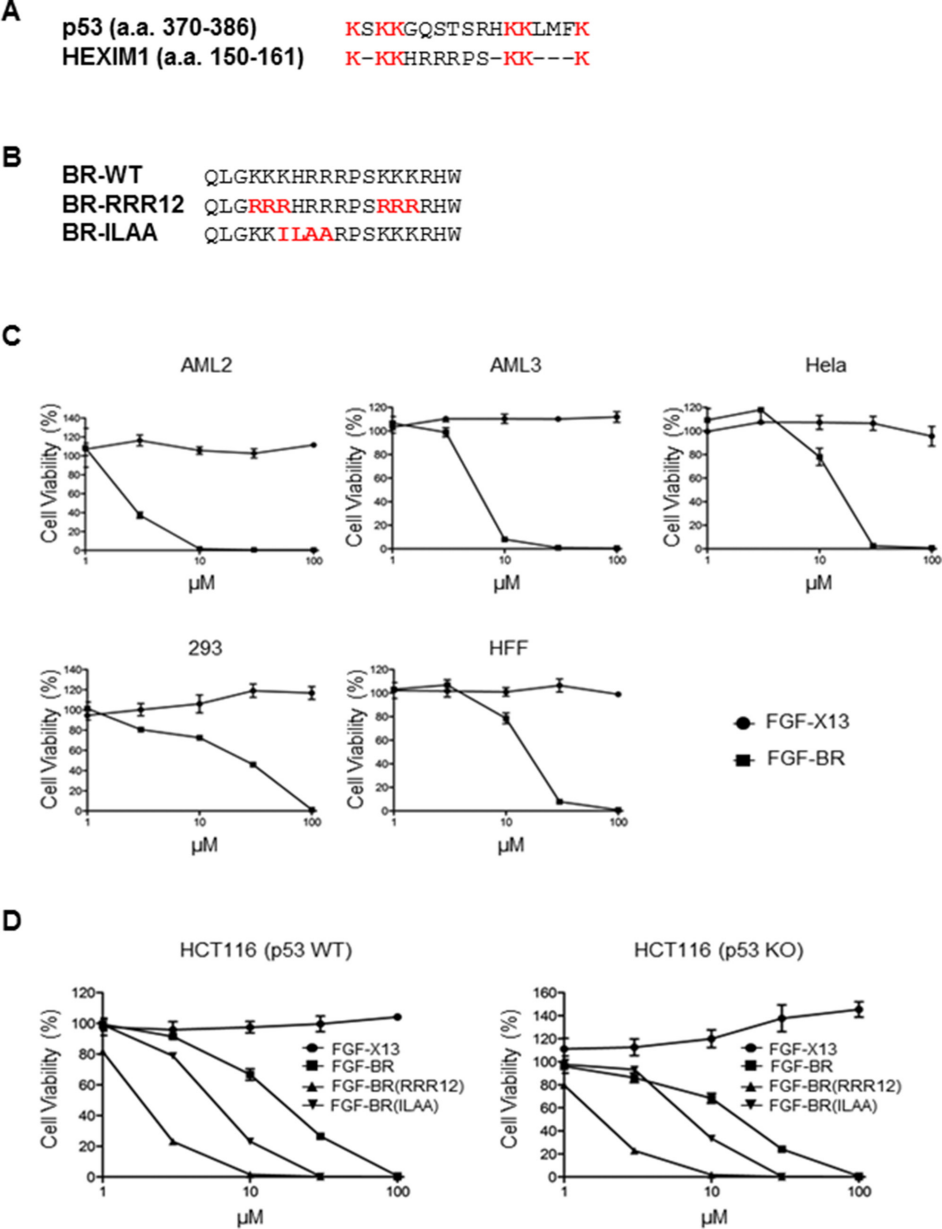
FGF-tagged BR peptide induces cell death (**A**) Alignment of the HDM2 ubiquitination sites between p53 (amino acids 370–386) and HEXIM1 (amino acids 150–161). The HDM2-ubiquitinated lysine residues are indicated in red. (**B**) Alignment of wild-type and mutant HEXIM1 BR peptides (RRR12 and ILAA). The mutated residues are indicated in red. (**C**) Cancer (AML2, AML3 and HeLa) and non-cancer (293 and HFF) cell lines were treated overnight with various concentrations of indicated FGF-fused peptides before cell viability was determined by Cell-Titer Glo assays. Cells treated with the FGF-X13 peptide were used as a negative control. (**D**) HCT116 (wild-type) and HCT116 (p53 KO) cells were treated with FGF-X13, FGF-BR, FGF-BR-RRR12 and FGF-BR-ILAA peptides at various concentrations overnight before cell viability assays were performed. Data representative of at least three independent experiments performed in triplicate were shown with values expressed as mean ± SD.

Since HDM2 can ubiquitinate both p53 and HEXIM1, we sought to determine the sequence similarity in HDM2 ubiquitination sites between p53 and HEXIM1. The high sequence similarity in location of the six lysine residues suggests that HEXIM1 peptide may activate p53, resulting in p53-dependent cell cycle arrest or cell death (Figure 1A). It is known that a commercial p53 activating peptide, FGF-p53, contains the p53 ubiquitination sites (a.a. 361–382) conjugated to a hydrophobic cell-membrane translocating peptide derived from Kaposi fibroblast growth factor (FGF) [19]. We generated a FGF-BR fusion peptide, in which the cell-penetrating FGF peptide was fused with HEXIM1 BR peptide containing the HDM2 ubiquitination residues (a.a. 147–164) (Figure 1B). We treated two acute myeloid leukemia (AML) cell lines, AML2 and AML3, with FGF-BR peptide to determine the effects of the peptide on cell viability. It has been reported that the p53 level in AML2 is significantly higher than that in AML3 [17]. Despite this, FGF-BR exhibited similar cytotoxicity on both cell lines, raising the possibility that the cell killing mediated by FGF-BR might not depend on p53 (Figure 1C). Similar result was obtained for human cervical cancer cells, HeLa cells, when treated with FGF-BR peptide (Figure 1C). Comparable cytotoxicity was also observed in normal cells, including HEK293 cells and human foreskin fibroblasts (HFFs) (Figure 1C), suggesting the cytotoxic effect of BR peptide occurs to all cell types once it is internalized into cells.

We tested the effects of FGF-BR peptide on HCT116 (p53 KO) cells and HCT116 (p53 WT) was used as a control cell line. FGF-BR induced cell death in both cell lines with similar potency, while the negative control peptide, FGF-X13, did not reduce cell viability in both cell lines (Figure 1D). We next generated a fusion peptide, FGF-BR-RRR12, in which all six HDM2 ubiquitinating lysine residues were mutated to arginine and could no longer be ubiquitinated by HDM2 (Figure 1B). If BR peptide induces cell death through the HDM2-p53 regulatory pathway, BR-RRR12 mutant is expected to lose its cytotoxic effects since HDM2 will continue to ubiquitinate p53 for degradation and inhibit cell death. Interestingly, the data suggests that FGF-BR-RRR12 peptide demonstrated stronger cellular toxicity when compared to the wild-type FGF-BR peptide (Figure 1D). Furthermore, we observed that the status of p53 had no effects on the potency of FGF-BR-RRR12 peptide, providing more evidence that the cell death caused by the BR peptide was not p53-dependent.

HEXIM1 is known as a negative regulator of P-TEFb through the association with 7SK snRNA [4]. The 7SK snRNA binding motif, KHRR, is present within the cytotoxic BR peptide. When KHRR was mutated to ILAA (Figure 1B), the mutant HEXIM1 could not associate with 7SK snRNA or inhibit P-TEFb activity [4]. We found that FGF-BR-ILAA peptide maintained its killing capacity in both HCT116 (p53 WT) and HCT116 (p53 KO) cell lines, indicating that P-TEFb might not be involved in the HEXIM1 BR-mediated cell killing (Figure 1D).

**Figure 2:**
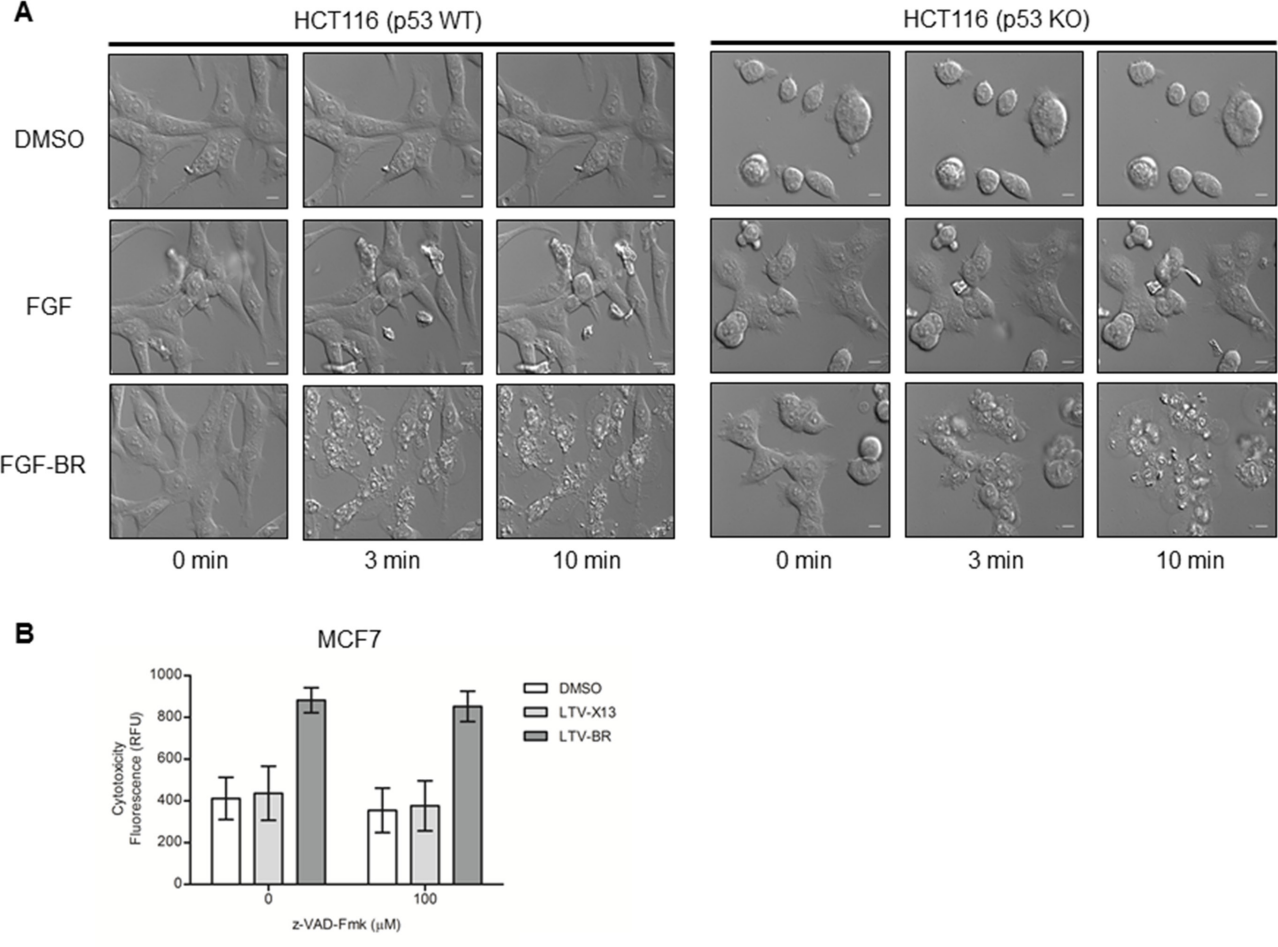
FGF-/LTV-tagged BR peptide induces rapid cell death independently of apoptosis (**A**) HCT116 (p53 WT) and HCT116 (p53KO) cells were cultured on glass slides overnight, treated with vehicle control (0.5% DMSO) or the indicated FGF peptides (30 μM). Time-lapse differential interference contrast (DIC) imaging highlights dynamic morphological changes in treated HCT116 cells by spinning disk confocal microscopy (Nikon). Representative DIC snapshots of treated HCT116 cells at indicated times were shown (*bar*, 100 μm). (**B**) MCF7 cells were incubated with or without the pan-caspase inhibitor z-VAD-Fmk (90 min) and then with indicated LTV-tagged peptides (30 μM) for thirty minutes at 37°C (LTV: breast cancer targeting peptide). Cells treated with LTV-X13 peptide or vehicles, DMSO (0.5%), were used as negative controls. Treated cells were subjected to the cytotoxicity assay as described in Methods. Data representative of at least three independent experiments performed in triplicate were shown with values expressed as mean ± SD.

We next investigated the mechanism of cell death induced by the HEXIM1 BR peptide. To monitor the real-time changes to the cells upon treatment with BR peptide, we examined the effect of FGF-BR on HCT116 (p53 WT) and HCT116 (p53 KO) cells in a live cell imaging setting. Cells treated with FGF peptide were included as a control. Within minutes, FGF-BR peptide rapidly induced drastic changes to the cell morphology with rupturing of the plasma membrane accompanied with damages to the nuclear membrane and abnormalities to the nucleolus in both cell types (Figures 2A and S1). No effects were detected when cells were treated with FGF peptide (Figure 2A). Similar observations were seen for MCF7 breast cancer cells treated with LTV-tagged BR peptide, which BR peptide is conjugated to a breast cancer-targeting peptide, LTV (data not shown). We rule out the possibility that HEXIM1-BR leads to apoptosis. In our study, BR-induced cytotoxicity occurred in minutes, whereas the duration of apoptosis is estimated from 12 to 24 hours [20]. In addition, the induction of cell death in LTV-BR-treated MCF7 cells could not be inhibited by a pan-caspase inhibitor z-VAD-Fmk (100 μM) (Figure 2B), indicating that HEXIM1-BR induced cell death is independent of apoptosis.

### HEXIM1 BR peptide induces rapid depolarization of mitochondrial membrane potential in a p53-dependent manner

**Figure 3:**
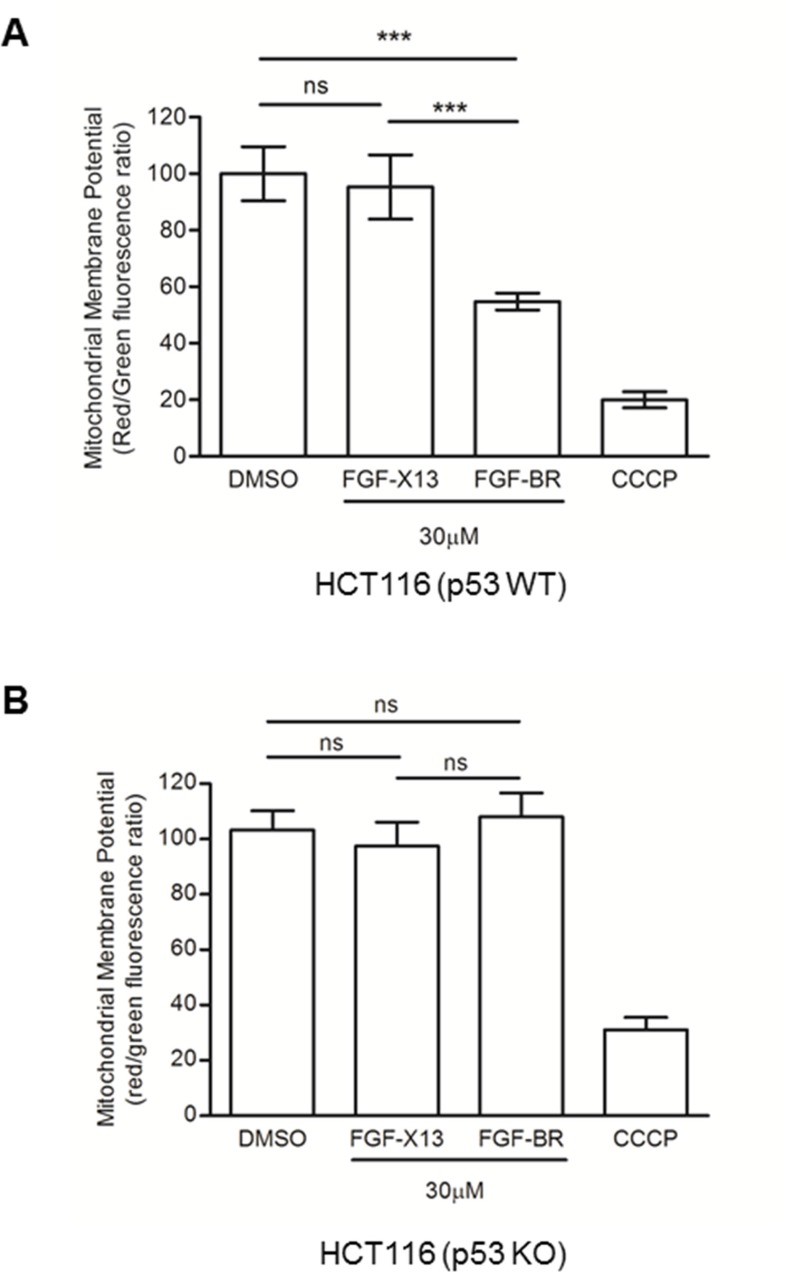
FGF-tagged BR peptide decreases mitochondrial membrane potential (**A**) HCT116 (p53 WT) and (**B**) HCT116 (p53 KO) cells were cultured in 96-well plates, treated with indicated FGF-tagged peptides (30 μM) for three minutes. A mitochondrial membrane depolarizer, carbonyl cyanide 3-chlorophenylhydrazone (CCCP), was used as a positive control. Cells treated with FGF-X13 peptide or vehicle, DMSO (0.5%), was used as negative controls. Treated cells were subjected to MMP measurement using JC-1 fluorescence-based assay for six independent experiments. Results were summarized as mean ± SD (***P < 0.0001; ns: not significant, Student's *t* test).

It has been reported that a commonly used cytotoxic basic peptide, KLA, initiates apoptotic cell death by disrupting the mitochondrial membrane potential (MMP) [21, 22]. It is possible that HEXIM1 BR utilizes similar approach to kill cells as it also contains many basic residues. We observed that FGF-BR-treated HCT116 (p53 WT) cells experienced rapid mitochondrial depolarization within three minutes (Figure 3A), which is similar to the time frame that morphological changes occurred upon addition of the FGF-BR peptide (Figure 2A). It is known that p53 translocates to the mitochondria and results in reduction of MMP in p53-mediated apoptosis [23]. Our study showed that the MMP of FGF-BR-treated HCT116 (p53 KO) cells did not decrease (Figure 3B) although these cells encountered rapid cytotoxicity similar to HCT116 (p53 WT) as mentioned earlier (Figure 2A). We reasoned that the depolarization of MMP induced by FGF-BR might not be essential to trigger rapid cell death. Collectively, our data supports that HEXIM1 BR peptide is likely to induce cell killing though an alternative pathway that acts independently of p53 and apoptosis.

### HEXIM1 BR peptide alters subcellular localization of NPM and reduces its protein expression

**Figure 4:**
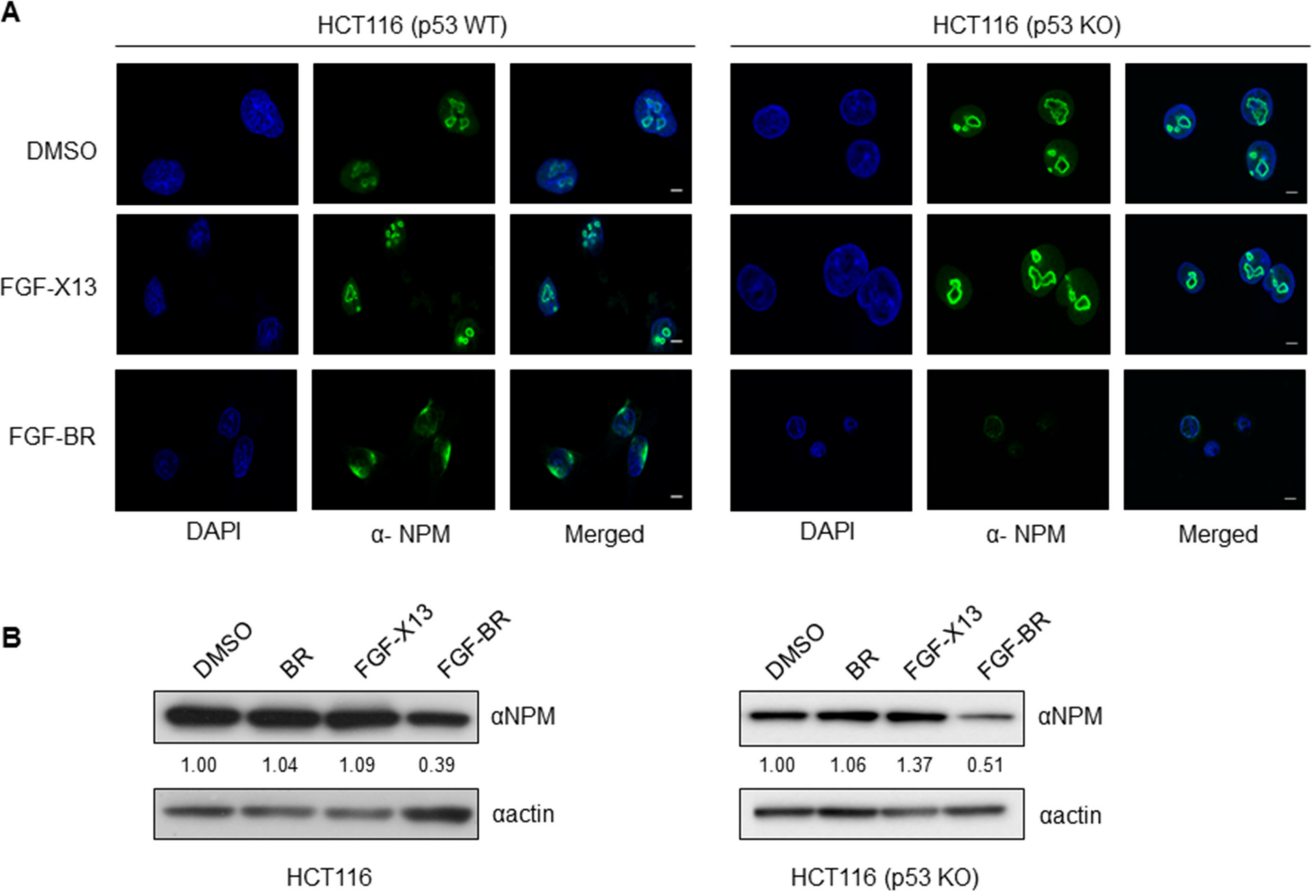
FGF-tagged BR peptide alters the sub-cellular localization and protein level of endogenous NPM (**A**) HCT116 (p53 WT) and HCT116 (p53KO) cells were cultured on glass slides overnight, treated with FGF-X13 or FGF-BR (30 μM). Cells treated with FGF-X13 peptide or vehicle, DMSO (0.5%), was used as controls. Treated cells were immunostained with an anti-NPM (green) antibody and analyzed by laser scanning confocal microscopy (Zeiss). Nuclei were visualized by DAPI. Representative fluorescent images were shown (*bar*, 10 μm). (**B**) HCT116 cells were plated overnight prior to addition of vehicle control (0.5% DMSO) or indicated peptides (30 μM) at 37°C. Lysates were subsequently harvested and subjected to western blotting with anti-NPM and anti-actin antibodies. The level of NPM protein was quantified as described in Materials and Methods.

NPM is an abundantly expressed nucleolar protein and a key regulator in ribosome biogenesis [13, 14]. The BR domain of HEXIM1 is known to contain a nucleolar localization signal. When BR was fused with yellow fluorescent protein (YFP), the BR-YFP was localized to the nucleolus [24]. In our previous study, we had identified NPM as a HEXIM1 binding protein partner and that the BR domain of HEXIM1 was required for NPM binding [12]. To investigate the effect of BR peptide on NPM, FGF-BR-treated HCT116 (p53 WT) and HCT116 (p53 KO) cells were immunostained with an anti-NPM antibody to examine the sub-cellular distribution of NPM. Normal nucleolar localization of NPM was observed in control experiments [Figure 4A, dimethyl sulfoxide (DMSO) and FGF-X13], but mislocalization of NPM was detected when cells were incubated with FGF-BR (Figure 4A, FGF-BR) in both cell types. Furthermore, we observed a reduction in NPM protein level in the FGF-BR treated HCT116 (p53 WT) and HCT116 (p53 KO) cells as compared to controls (Figure 4B). Various post-translational modifications of p53, namely phosphorylation and acetylation, have been shown to stabilize and activate p53 in response to cellular stress [25, 26]. We then determined the expression levels of phosphorylation of p53 on Ser15 and acetylation of p53 on Lys382 and found that they remained unchanged in HCT116 (p53 WT) cells when treated with FGF-BR peptide (data not shown), suggesting a p53-independent pathway to trigger cell death. These results demonstrate that the BR peptide may interfere with protein translation/ribosome biosynthesis by disrupting sub-cellular localization of NPM and decreasing its expression, hence compromising its normal function.

### LTV-BR fusion peptide kills breast cancer cells selectively

**Figure 5:**
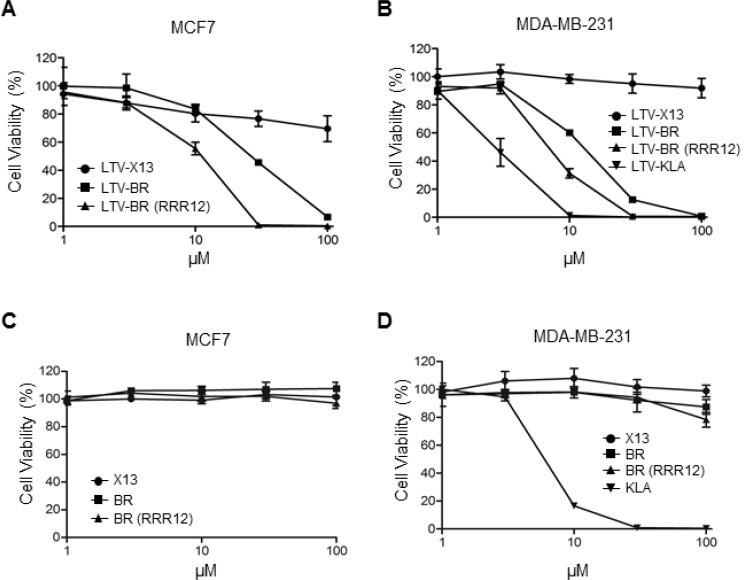
LTV-fused BR peptide decreases cell viability in breast cancer cells Two breast cancer cell lines, (**A**) MCF7 and (**B**) MDA-MB-231 were treated with indicated LTV-fused peptides at different concentrations overnight before cell viability assays were performed. Effects of the indicated un-tagged peptides on the viability of (**C**) MCF7 and (**D**) MDA-MB-231 cells were analyzed by cell viability assays. The untagged X13 was used as a negative control. Data representative of at least three independent experiments performed in triplicate were shown with values expressed as mean ± SD.

To explore the potential use of the cytotoxic HEXIM1 BR peptide in cancer therapy, we fused a breast cancer targeting peptide LTV, to HEXIM1 BR and BR-RRR12 to generate the fusion peptides namely, LTV-BR and LTV-BR-RRR12. Both peptides exhibited anti-proliferative effects against two breast cancer cell lines, MCF7 and MDA-MB-231, while cells remained viable when treated with LTV-X13 control peptide (Figure 5A and 5B). Although MDA-MB-231 cells are triple-negative with the absence of expression of oestrogen receptor, progesterone receptor and HER2, LTV-BR and LTV-BR-RRR12 elicited similar anti-cancer activity as compared to MCF7 cells (Figure 5B). In addition, untagged control X13 and the two BR peptides had no effect on cell viability (Figure 5C and 5D). To define the active region of the BR peptide, we generated a series of truncated BR peptides based on the stretches of basic residues found in the BR peptide sequence. These truncated peptides were fused to LTV and introduced to MDA-MB-231 cells. The results suggest that only the region encompassing the second and third stretch of basic residues (HRRRPSKKKRHW) is more critical in exerting cytotoxic activity as compared to the first stretch of basic residues (Figure S2). We proceeded to replace stretches of basic residues to alanine residues in the active region of BR and obtained similar conclusion on the importance of the same stretches of basic residues (HRRRPSKKKRHW) as essential for inducing cytotoxicity (Figure S3). Nonetheless, all three stretches of basic residues are required to exhibit the maximum cytotoxic effect against MDA-MB-231 cells.

KLA peptide, a cytotoxic peptide, was often fused with a cancer-targeting peptide or conjugated to antibodies recognizing cancer cells to exert cell-killing effect in anti-cancer therapy [27, 28]. We generated LTV-KLA peptide and compared its effect to that of LTV-BR peptides. LTV-KLA exhibited stronger inhibition on the viability of MDA-MB-231 cells (Figure 5B). However, we found that the untagged KLA peptide alone showed non-specific killing on MDA-MB-231 cells, while BR- or BR-RRR12-treated cells remained highly viable (Figure 5D). LTV-KLA exhibited non-specific cell killing on non-breast cancer cell lines, CHO (Chinese hamster ovary) and OPM-2 (multiple myeloma) cells, while little or no effects were observed in the LTV-BR-treated cells (Figure S4A and S4B). Similar observations were seen for normal human fibroblasts HFF and WI-38, indicating the specificity of LTV-BR towards breast cancer cell lines but not LTV-KLA (Figure S4C and S4D). Thus, the use of KLA as the toxic load in anti-cancer therapy is likely to be questioned on its specificity. As untagged HEXIM1 BR peptide is unable to kill cells when it is not fused with any cell-penetrating or targeting peptides, our data suggest that HEXIM1 BR peptide may be a safer alternative as compared to KLA, for the development of anti-cancer therapeutics.

**Figure 6:**
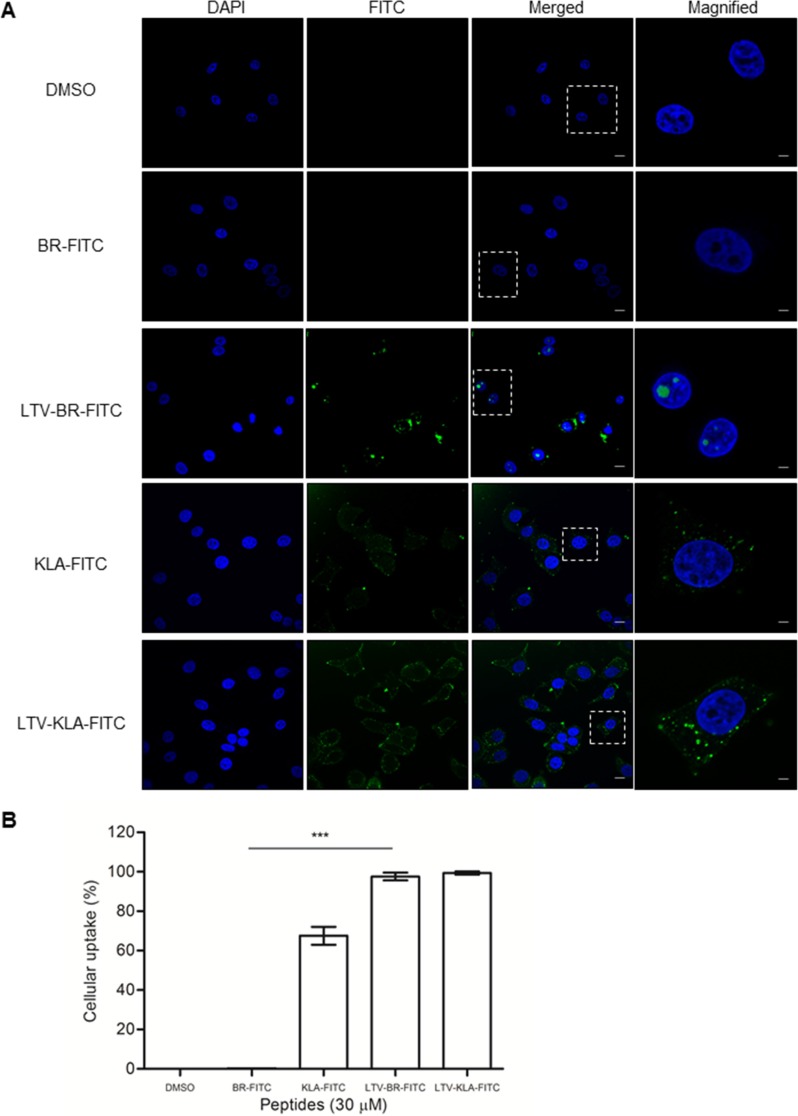
The un-tagged HEXIM1 BR peptide fails to enter cells by itself (**A**) MCF7 cells were cultured on glass slides overnight before incubation with the indicated FITC-labeled peptides (30 μM) for 30 min and subsequently analyzed by laser scanning confocal microscopy. Nuclei were visualized by DAPI. Cells treated with vehicle, DMSO (0.5%) were used as control. Representative fluorescent images are shown (*bar*, 10 μm). (**B**) The FITC-positive cells were quantified by flow cytometry. Data represent percentage of fluorescence-positive cells in total cell population. Results were summarized as mean ± SD of three independent experiments (***P < 0.0001, Student's *t* test).

The HEXIM1 BR peptide did not cause any cytotoxic effect when it was not fused with a cell-penetrating (*i.e*. FGF) or cancer cell targeting (*i.e.* LTV) peptide. It is possible that the untagged HEXIM1 BR peptide may fail to internalize into cells by itself without specific guidance. To test this hypothesis, we treated MCF7 cells with fluorescent-labeled BR, LTV-BR, KLA, and LTV-KLA peptides and examined the intracellular distribution of these peptides using confocal microscopy. No fluorescent signal was detected in the DMSO vehicle control as well as BR peptide (Figure 6A). LTV-BR was readily internalized into MCF7 cells and distributed in cytoplasm and nuclei (Figure 6A). It was noted that its strong fluorescent signals were primarily localized in the nucleoli (Figure 6A, LTV-BR-FITC). Detection of fluorescent signals in KLA-treated cells helps to explain the non-specific cytotoxicity induced by KLA peptide (Figure 6A), while no fluorescent signal was observed in HEXIM1 BR-treated cells, indicating that the BR peptide could not enter the cells by itself (Figure 6A, BR-FITC). Cells treated with LTV-KLA demonstrated that the sub-cellular localization of the peptide was observed mainly in the cytoplasm (Figure 6A). The different distribution of LTV-BR and LTV-KLA suggests that BR and KLA may utilize different mechanisms for cell killing. Flow cytometric analysis was also performed to quantify the amount of internalized fluorescent peptide in MCF7 cells. LTV peptide directed the uptake of almost 100% of LTV-fused peptides (i.e. LTV-BR and LTV-KLA) into the breast cancer cell line (Figure 6B). Approximately 65% of KLA-FITC-treated MCF7 cells internalized KLA-FITC, whereas there was no entry of BR-FITC into MCF7 cells (Figure 6B). These results clearly demonstrate the safety feature of HEXIM1 BR peptide when compared to the non-specific cytotoxic KLA peptide. LTV assisted in the cellular internalization of HEXIM1 BR peptide into its target cells and the nucleolar localization of the fusion peptide might be subsequently guided by BR peptide.

## DISCUSSION

In this study, we report the potential use of a novel cytotoxic peptide derived from the BR of HEXIM1. When fused with a cell-penetrating (FGF) or a breast cancer-targeting (LTV) peptide, the HEXIM1 BR fusion peptide induces rapid cell death *in vitro* through an apoptosis- and p53-independent mechanism. This is accompanied with the disruption of sub-cellular localization and reduction in the protein level of NPM, which compromise ribosome biogenesis and limit protein synthesis. In addition to its role in protein translation, NPM is required to maintain DNA integrity in cells. Knockout of NPM results in accumulation of DNA damage, which clearly indicates the essential role of NPM in cell proliferation and survival [29]. We showed that HEXIM1 BR rapidly depolarizes MMP only in presence of p53. This is unlikely a major factor leading to cell death as cells without p53 are also subjected to cytotoxicity induced by HEXIM1 BR peptide. Since HEXIM1 BR peptide severely damages the fundamental biological function essential to all cell types, we reason that the peptide could be utilized in the development of novel treatment against drug-resistant cancer guided by a specific cancer targeting peptide.

In addition, cancer cells tend to evade apoptosis by overexpressing anti-apoptotic proteins like Bcl-2 [30, 31] or down regulating pro-apoptotic proteins [32]. The duration of apoptosis is estimated between 12 to 24 hours [20]. In our study, we demonstrated that treatment with FGF-BR peptide rapidly induces cell death in minutes (Figure 2A). Moreover, the morphological changes seen in FGF-BR treated HCT116 cells (Figure S1) do not resemble the characteristics of cells dying by apoptosis which include membrane blebbing and formation of apoptotic bodies [33]. Cell swelling and subsequent rupturing of the plasma membrane followed by rapid lysis of the cells observed were descriptive of necrosis instead (Figures 2A and S1) [34]. It is unlikely that the conjugated HEXIM1-BR peptide triggered apoptosis in the treated cells based on the time frame observed for inducing cell death and the ability to kill cells despite the presence of the caspase inhibitor (Figure 2B). Hence, using this BR peptide provides an attractive approach to eliminate cancer cells that have a defective apoptotic pathway.

Interestingly, we showed that the BR peptide alone did not reduce cell viability, as compared to the KLA peptide (Figure 5D). The effect of adding KLA peptide has been shown to induce significant non-specific cell death in several cell lines [27, 28, 35]. Our data also suggested that KLA alone could be internalized into cells and decrease cell viability (Figure 6A). Therefore, KLA peptide may not be an ideal toxin used to generate a therapeutic fusion peptide due to its non-specific toxicity. The use of toxic peptides in generation of antibody-drug conjugate (ADC) has been shown previously with the use of KLA [27, 36]. It is predictable that a portion of the ADCs will be broken down before reaching the target cancer cells, even though non-cleavable linkers may be used to generate these conjugates. As such, KLA peptides may be released from the conjugates, resulting in an off-target killing of normal cells/tissues. Treating cells with HEXIM1 BR alone had minimal effects to the cells as the peptide could not be internalized by itself (Figure 6A). We believe that the BR peptide can provide a better choice than KLA peptide in the generation of a safer ADC in anti-cancer treatment and efforts to conjugate the BR peptides to other potential monoclonal antibodies targeting against cancer are ongoing.

Taken together, we demonstrate the potential use of the HEXIM1 BR peptide in cancer therapy. Importantly, our data show the safety aspect of HEXIM1 BR peptide compared to other cytotoxic peptides. The HEXIM1 peptide induces rapid and potent cytotoxicity only when the peptide is internalized within the cells, which supports its further consideration as a promising therapeutic approach in treatment of any forms of cancers by fusing to different targeting peptides which recognize specific cancer cells.

## MATERIALS AND METHODS

### Cell lines

Human cell lines including HeLa, HEK293, MCF7, MDA-MB-231, CHO-K1, OPM-2, and WI-38 were obtained from American Type Culture Collection. AML2 and AML3 cells were purchased from Deutsche Sammlung von Mikroorganismen und Zellkulturen. HCT116 *p53*^+/+^ and *p53*^−/−^ cells were kindly given by Dr. Bert Vogelstein [37]. Primary human foreskin fibroblasts (HFF) were obtained from Dr. Mark Stinski [38]. HCT116, HCT116 (p53 KO), HeLa, 293, HFF, MCF7, CHO-K1, and WI-38 cells were cultured in Dulbecco's Modified Eagle Medium (DMEM) supplemented with 10% fetal bovine serum (FBS) and 1% penicillin/streptomycin (Gibco). MDA-MB-231 and OPM-2 cells were cultured in RPMI-1640 (Gibco) containing 10% FBS and 1% penicillin/streptomycin. All cells were routinely maintained in a 37°C incubator with 5% CO_2_.

### Peptide synthesis

All peptides used in this study were chemically synthesized and purified by high performance liquid chromatography with > 98% purity (First Base, Singapore). Their sequences are available in the Table S1. Stock solutions were obtained by reconstituting the powder in sterile water or 50% DMSO and stored at −80°C.

### Immunoblotting analysis

Cells were lysed in lysis buffer [50 mM Tris-HCl, (pH 7.5), 150 mM NaCl, 1% NP40, 0.5% sodium deoxycholate, Protease Inhibitor tablet (Roche)] and used for western blotting. Western blotting was carried out as previously mentioned [9]. The primary antibodies used in this study included anti-NPM (Invitrogen) and anti-actin (Millipore). The film of western blot was scanned, and the protein bands were quantified by the GS-800 densitometer (Bio-Rad). The protein level of NPM was quantified after normalizing with the loading control, actin.

### Cell viability and cytotoxicity assays

Cells were plated in clear-bottomed white walled 96-well plates (Corning) and incubated overnight. Cells were treated with indicated peptides in 1% FBS-containing media for overnight or indicated timings at 37°C. Cell viability was measured with CellTiter-Glo reagent (Promega) according to the manufacturer's instructions. For cytotoxicity assay, cells were plated in black walled 96-well plates (Corning) to allow them to adhere overnight. Upon treatment with a pan-caspase inhibitor, z-VAD-Fmk (100 μM) (Sigma) and subsequent treatment with LTV-tagged peptides, cytotoxicity induced was determined by CellTox cytotoxicity assay (Promega) 30 minutes after addition of peptides according to the manufacturer's instructions. Luminescence was determined using an Infinite 200 multiplate reader (Tecan).

### Measurement of MMP

The cationic fluorescent dye 1, 1′, 3, 3′-tetraethylbenzamidazolocarbocyanin iodide (JC-1) (Invitrogen) was utilized for MMP measurement. JC-1 was dissolved in DMSO (200 μM). Peptide-treated cells in 96-well deep sided, clear bottom, dark sided microplates were incubated with media containing JC-1 for 30 min at 37°C and then washed twice with warm PBS. Changes in MMP were determined using a multiplate spectrofluorometer (Tecan) [excitation: 475 nm; emission: 530 nm (green); emission: 590 nm (red)]. The decrease in the ratio of red to green fluorescence was used to determine relative mitochondrial depolarization.

### Flow cytometry

MCF7 cells were plated on 6-cm culture dish to allow overnight adherence. FITC-labeled peptides (30 μM) were added to the cells, incubated for 30 min at 37°C, and subsequently washed three times with PBS. The cells were then trypsinized, collected by centrifugation, and finally resuspended in 500 μl ice-cold 2% FBS-containing PBS for flow cytometry analysis.

### Immunofluorescence and confocal microscopy

For staining with anti-NPM, cultured cells were fixed in 10% neutral buffered formalin (Sigma) for 10 min, and then in methanol for 10 min, washed in PBS and incubated with blocking buffer (PBS containing 0.5% bovine serum albumin) for 1 hour prior to incubation with a mouse anti-NPM antibody (Invitrogen) in blocking buffer for overnight at 4°C. Cells were then incubated with Alexa Fluor 488-conjugated secondary antibody (Jackson Immuno Research Laboratories) for 1 hour, washed three times with PBS and counterstained with 4′,6-diamidino-2-phenylindole (DAPI) -containing mounting solution (Vectashield). Stained cells were examined with a LSM 510 confocal microscope using a 63 × objective lens (Zeiss).

To determine the ability of FITC-labeled peptides to enter the cells and to visualize intracellular distribution of the peptides, MCF7 cells were plated on 4-chamber glass cover slides (Lab-Tek) to adhere overnight, incubated with FITC-labeled peptides (30 μM) for 30 min, and then washed three times with PBS before being fixed and mounted with DAPI-containing mounting solution (Vectashield). Images were acquired using a Nikon A1R confocal laser scanning microscope equipped with a 60 × oil-immersion objective lens (SBIC-Nikon Imaging Centre).

### Statistical analysis

All experiments were performed independently for at least three times. All statistical analyses for comparison between two groups were performed with two-tailed unpaired student's *t*-test using the Prism 5.01 (GraphPad Software).

## SUPPLEMENTARY MATREIALS FIGURES AND TABLES






