# Supplementary material for: Use of a novel cytotoxic HEXIM1 peptide in the directed breast cancer therapy
Source: Oncotarget. 2015 Dec 29;7(5):5483–94. doi: 10.18632/oncotarget.6794 (PMC4868700; doi:10.18632/oncotarget.6794)
Supplement: Supplementary file 1 [file oncotarget-07-5483-s001.pdf]

Use of a novel cytotoxic HEXIM1 peptide in the directed breast cancer therapy

Supplementary Materials

Supplementary Figure S1: FGF-BR peptide induced rapid cytotoxicity to cells. HCT116 cells were plated on 4-chamber glass cover slides (Lab-Tek) to attach overnight at appropriate density to achieve 50% confluence the next day. FGF-tagged X13 or BR peptides (30 µM) was added to the cells. Microscopic time-lapse images were taken every 30 seconds for 30 mins at 37°C. Images were acquired on a Nikon Eclipse Ti inverted microscope, equipped with a Plan Apo VC 60X/1.4 NA DIC oil immersion objective lens, the Perfect Focus System (to maintain focus during time-lapse experiments), a motorized stage, an X-cite 120PC Q metal halide fluorescence lamp (Lumen Dynamics), a pE-100 transmitted lamp (cooLED) and a Coolsnap HQ2 CCD camera (Photometrics). Samples were incubated in a stage-top incubator with humidified chamber (Live-Cell Instruments) at 37°C and 5% CO_2. The multidimensional data acquisition was controlled by MetaMorph software (Molecular Devices).

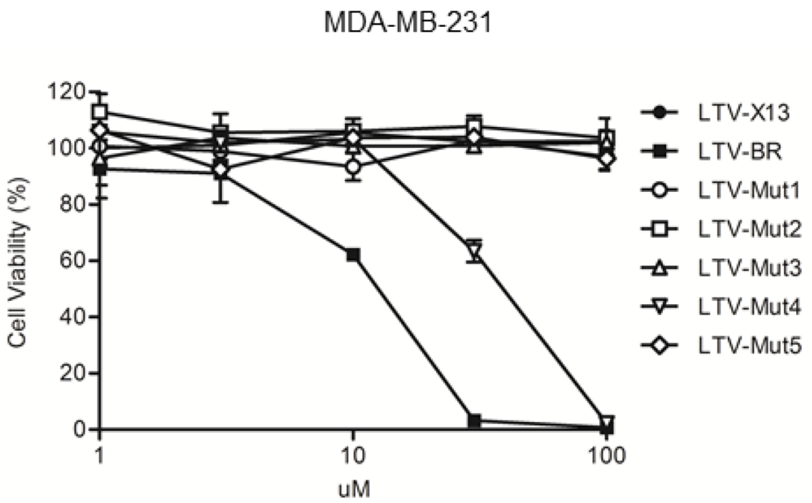

Name	Sequence	Cell Killing Potency
LTV-BR	LTVSPWYGCGQLGKKKHRRRPSKKKRHW	++
LTV-Mut1	LTVSPWYGCGQLGKKKH-----------	-
LTV-Mut2	LTVSPWYGCGQLGKKKHRRRPS------	-
LTV-Mut3	LTVSPWYGCG----------PSKKKRHW	-
LTV-Mut4	LTVSPWYGCG------HRRRPSKKKRHW	+
LTV-Mut5	LTVSPWYGCG------HRRRPS------	-

Supplementary Figure S2: LTV-tagged BR peptide required all three stretches of basic residues to establish full potency in cell killing. MDA-MB-231 breast cancer cells were treated with indicated truncated LTV-fused peptides at various concentrations overnight before cell viability assays were performed. Cells treated with LTV-X13 was used as a negative control. Data representative of at least three independent experiments performed in triplicate were shown with values expressed as mean ± SD.

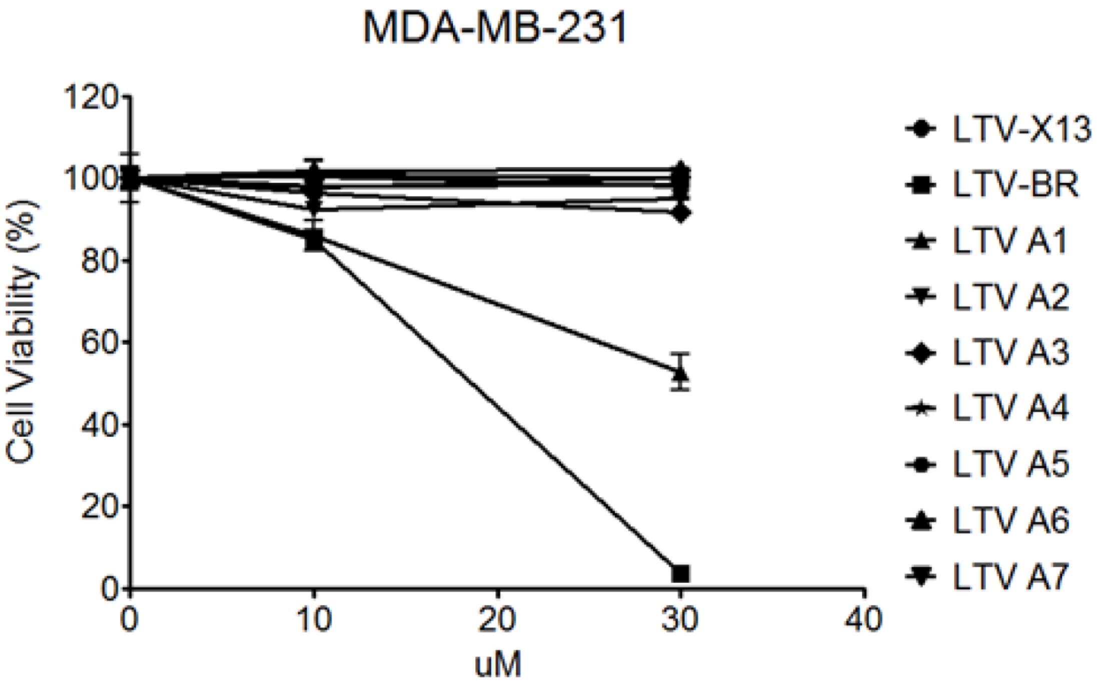

Name	Sequence	Cell Killing Potency
LTV-BR	LTVSPWYGCGQLGKKKHRRRPSKKKRHW	++
LTV-BR(A1)	LTVSPWYGCGQLGAAAHRRRPSKKKRHW	+
LTV-BR(A2)	LTVSPWYGCGQLGKKKHAAAPSKKKRHW	-
LTV-BR(A3)	LTVSPWYGCGQLGKKKHRRRPSAAAAHW	-
LTV-BR(A4)	LTVSPWYGCGQLGAAAHAAAPSKKKRHW	-
LTV-BR(A5)	LTVSPWYGCGQLGAAAHRRRPSAAAAHW	-
LTV-BR(A6)	LTVSPWYGCGQLGKKKHAAAPSAAAAHW	-
LTV-BR(A7)	LTVSPWYGCGQLGAAAHAAAPSAAAAHW	-

Supplementary Figure S3: Alanine residue substitution in the middle and C-terminal regions of the HEXIM1 BR peptide abolished the cytotoxic activity of LTV-BR. MDA-MB-231 breast cancer cells were treated with indicated alanine-substituted LTV-fused peptides at 10 µM and 30 µM overnight before cell viability assays were performed. Data representative of at least three independent experiments performed in triplicate were shown with values expressed as mean ± SD.

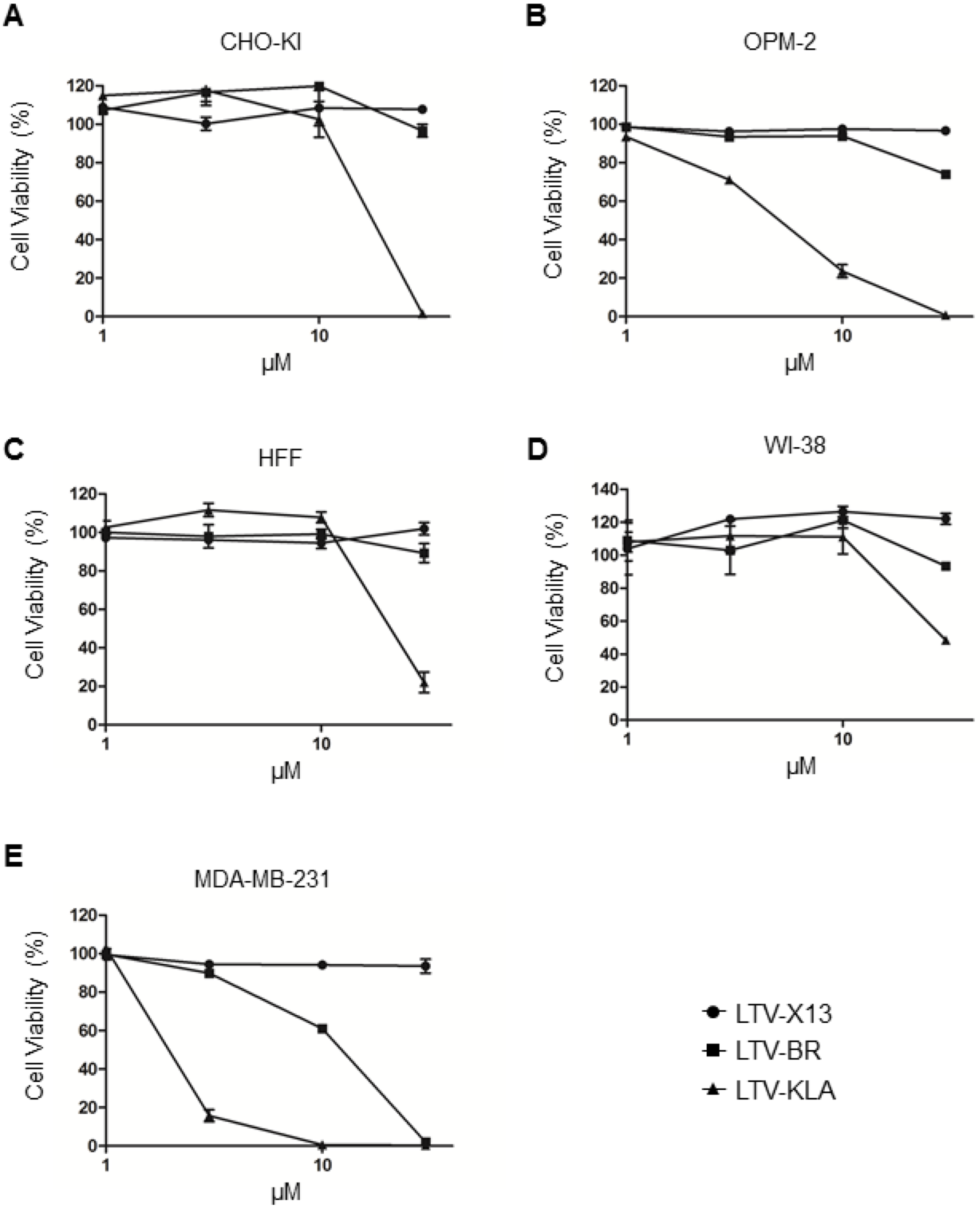

Supplementary Figure S4: LTV-tagged KLA peptide exhibits non-specific killing on non-breast cancer cell lines and normal human fibroblasts. CHO-K1 (Chinese hamster ovary), OPM-2 (multiple myeloma) cells, HFF (primary human foreskin fibroblasts) and WI-38 (primary human lung fibroblasts) were treated with indicated LTV-fused peptides at various concentrations overnight before cell viability assays were performed. Cells treated with LTV-X13 was used as a negative control. Data representative of at least three independent experiments performed in triplicate were shown with values expressed as mean ± SD.

Supplementary Table S1: Sequences of synthesized peptides

Peptide Name	Sequence
X13	MPFSTGKRIMLGE
BR	QLGKKKHRRRPSKKKRHW
KLA	KLAKLAKKLAKLAK
LTV-X13	LTVSPWYGCGMPFSTGKRIMLGE
LTV-BR	LTVSPWYGCGQLGKKKHRRRPSKKKRHW
LTV-BR (RRR12)	LTVSPWYGCGQLGRRRHRRRPSRRRRHW
LTV-KLA	LTVSPWYGCGKLAKLAKKLAKLAK
BR-FITC	QLGKKKHRRRPSKKKRHW-Lys(FITC)
LTV-BR-FITC	LTVSPWYGCGQLGKKKHRRRPSKKKRHW-Lys(FITC)
KLA-FITC	KLAKLAKKLAKLAK-Lys(FITC)
LTV-KLA-FITC	LTVSPWYGCGKLAKLAKKLAKLAK-Lys(FITC)
FGF-X13	AAVALLPAVLLALLAPMPFSTGKRIMLGE
FGF-BR	AAVALLPAVLLALLAPQLGKKKHRRRPSKKKRHW
FGF-BR(RRR12)	AAVALLPAVLLALLAPQLGRRRHRRRPSRRRRHW
FGF-BR(ILAA)	AAVALLPAVLLALLAPQLGKKILAARPSKKKRHW
